# Radiosensitizing effect of galunisertib, a TGF-ß receptor I inhibitor, on head and neck squamous cell carcinoma in vitro

**DOI:** 10.1007/s10637-021-01207-1

**Published:** 2022-01-05

**Authors:** Bernhard J. Jank, Teresa Lenz, Markus Haas, Lorenz Kadletz-Wanke, Nicholas J. Campion, Julia Schnoell, Gregor Heiduschka, Karin Macfelda

**Affiliations:** 1 Department of Otorhinolaryngology, Head and Neck Surgery, Medical University of Vienna, Waehringer Guertel 18-20, 1090 Vienna, Austria; 2 Department of Biomedical Research, Medical University of Vienna, Waehringer Guertel 18-20, 1090 Vienna, Austria

**Keywords:** Head and neck squamous cell carcinoma, Galunisertib, Radiosensitivity, TGF-ß

## Abstract

*Background*. Resistance to radiation therapy poses a major clinical problem for patients suffering from head and neck squamous cell carcinoma (HNSCC). Transforming growth factor ß (TGF-ß) has emerged as a potential target. This study aimed to investigate the radiosensitizing effect of galunisertib, a small molecule TGF-ß receptor kinase I inhibitor, on HNSCC cells in vitro. *Methods*. Three HNSCC cell lines were treated with galunisertib alone, or in combination with radiation. Of those three cell lines, one has a known inactivating mutation of the TGF-ß pathway (Cal27), one has a TGF-ß pathway deficiency (FaDu) and one has no known alteration (SCC-25). The effect on metabolic activity was evaluated by a resazurin-based reduction assay. Cell migration was evaluated by wound-healing assay, clonogenic survival by colony formation assay and cell cycle by FACS analysis. *Results*. Galunisertib reduced metabolic activity in FaDu, increased in SCC-25 and had no effect on CAL27. Migration was significantly reduced by galunisertib in all three cell lines and showed additive effects in combination with radiation in CAL27 and SCC-25. Colony-forming capabilities were reduced in SCC-25 by galunisertib and also showed an additive effect with adjuvant radiation treatment. Cell cycle analysis showed a reduction of cells in G_1_ phase in response to galunisertib treatment. *Conclusion*. Our results indicate a potential antineoplastic effect of galunisertib in HNSCC with intact TGF-ß signaling in combination with radiation.

## Background

Head and Neck Squamous Cell Carcinoma (HNSCC) is the seventh most common type of cancer worldwide, affecting more than 5.5 million people, causing over 380,000 deaths every year [1–3]. HNSCCs represents a group of tumors that can arise from the mucosa of the nasal or oral cavity, the pharynx or larynx. Despite multidisciplinary treatment approaches, advanced HNSCC has a 5-year overall survival rate of only ~ 50% [4]. Radiotherapy is an important cornerstone in the treatment of HNSCC patients, either as adjuvant therapy after surgery or as definitive radiochemotherapy in patients unfit for surgery or with locally advanced disease. Resistance to radiation is therefore particularly associated with a poor prognosis and represents a major clinical problem [5]. Strikingly, only the epidermal growth factor receptor antibody cetuximab has been approved as a radiosensitizing agent in the last 50 years [6].

Radioresistance is defined as either no or only partial response of the tumor to radiation therapy or early recurrence within a few weeks after initial response [5]. The biological mechanisms are complex and involve, among others, the Transforming Growth Factor (TGF) pathway with TGF-ß as one of its key members [7]. Although TGF-ß has tumor-suppressing functions in healthy cells and most early-stage cancers, its activation in late-stage disease can promote tumorigenesis [8]. Notably, increased TGF-ß expression has been found in 80% of HNSCC and TGF-ß expression levels correlate with more advanced disease and worse patient survival [9].

The experimental cancer drug galunisertib (LY2157299) is a small molecule inhibitor of the TGF-ß receptor I and has shown antineoplastic effects in various cancer entities in vitro and in vivo [10]. Furthermore, galunisertib is currently being tested in cancer clinical trials for several different cancers, highlighting the expected potential of this novel drug [11]. To date, however, no data exist on the effect of galunisertib on HNSCC. The aim of this study was, therefore, to preclinically evaluate the effect of galunisertib in single- and combination treatment with radiation on head and neck squamous cell carcinoma in vitro.

## Methods

### Cells and reagents

HNSCC cell lines (FaDu, SCC-25 and Cal 27) were purchased from American Type Culture Collection (ATCC, Manassas, Virginia, USA) and cultured in Dulbecco's Modified Eagle Medium supplemented with 10% FBS (Life Technologies) and 1% Penicillin–Streptomycin (Thermo Fisher, Waltham, Massachusetts, U.S.) on standard cell-culture plastic. Cells were cultured under standard culture conditions (37 °C in 5% CO2) and passaged with 0.05% trypsin/EDTA at 80% confluency. Galunisertib (LY2157299) was purchased from Selleck Chemicals (Houston, Texas, USA), dissolved in DMSO as stock solution following the manufacturer’s instructions and stored at -20 °C until use. The final dilution in culture medium was kept below 0.16% DMSO concentration. The same concentration of DMSO was added in untreated controls as a vector control.

### Irradiation

Cell lines were irradiated at a dose of 1.0 Gy/min at a fixed-focus object distance of 45.5 cm. A 200 kV YXLON Maxishot X-ray unit (Yxlon International X-ray GmbH, Hamburg, Germany) with a tube current of 20 mA and a focus size of 5.5 mm, 4 mm Al and 0.6 mm Cu filter served as the radiation source.

### Metabolic activity assay

To evaluate metabolic activity, 6 × 10^3^ cells were seeded into single wells of a 96-well plate. Cells were allowed to attach for 24 h and were subsequently exposed to either radiation, galunisertib, a combination of both or 0.16% DMSO which served as the control. For combination experiments, cells were exposed to different doses of irradiation, ranging from 2 to 8 Gy. Subsequently, metabolic activity was evaluated using a colorimetric resazurin-based redox assay as described previously [12]. In brief, resazurin sodium salt stock at a concentration of 570 µM was diluted at 1:10 v/v in the cell culture medium and cells were exposed for 60 min. Absorbance was measured at 570 nm using a microplate reader (Tecan Spark ®, Tecan Group Ltd., Maennedorf, Switzerland).

### Wound-healing assay

15 × 10^5^ cells of each cell line were seeded in each well of a 24 well plate. After cells reached 95–100% confluency, they were exposed to either galunisertib, radiation or a combination of both. DMSO-treated cells served as a control group. A handmade scratch was produced with a 200 µl pipette tip. The scratch was photographed at 0 h and 24 h using a microplate reader (Tecan Spark ®, Tecan Group Ltd., Maennedorf, Switzerland) and measured using ImageJ for further analysis.

### Colony formation assay

Colony formation assays were performed as described previously [13]. In brief, 5 × 10^2^ (CAL27), 6 × 10^2^ (FaDu) or 15 × 10^2^ (SCC-25) cells were plated in 6‐well plates and incubated for 24 h. Cells were then treated with galunisertib and/or irradiated with 4 Gy. After 72 h, the drug‐containing medium was replaced by a drug-free medium. After further 10 days, cells were washed with phosphate-buffered saline and photographed using a microplate reader (Tecan Spark ®, Tecan Group Ltd., Maennedorf, Switzerland). Colonies consisting of more than 50 cells were regarded as survivors and automatically counted using ImageJ. The surviving fractions were normalized to untreated controls.

### Flow cytometry

HNSCC cell lines were seeded at 1 × 10^6^ per well in 6-well plates. Cells were treated with either galunisertib, radiation, a combination of both or DMSO control 24 h after seeding. After 72 h of incubation, the cell cycle was analysed using DAPI staining and fluorescence-activated cell sorting (FACS) analysis (LSR Fortessa, BD Bioscience, USA).

### Statistical analysis

All results represent three independent experiments and are reported as mean ± SD. Statistical analysis was performed using GraphPad Prism for Mac Version 8 (GraphPad Software, LLC.). Statistical significance of differences between groups was determined using two-way-ANOVA and Tukey multiple comparison testing. A P-value below 0.05 was considered significant. Combinatory drug effects were defined using the Bliss independence method based on the assumption of independent drug mechanisms. In brief, the combined effect (E_T_) expressed as the surviving fraction is modelled as the product of the individual effects with drug “A” (E_A_) and “B” (E_B_), computed by: E_T_ = E_A_ x E_B_ [14]. If the drug combination is similar to the expected combined effect (E_AB_ = E_T_), then the combination would be additive, if it is less than expected (E_AB_ < E_T_) it would be synergistic and if it is greater (E_AB_ > E_T_) than it would be antagonistic [15].

## Results

### Galunisertib shows heterogeneous effects on metabolic activity in HNSCC cell lines

**Fig. 1:**
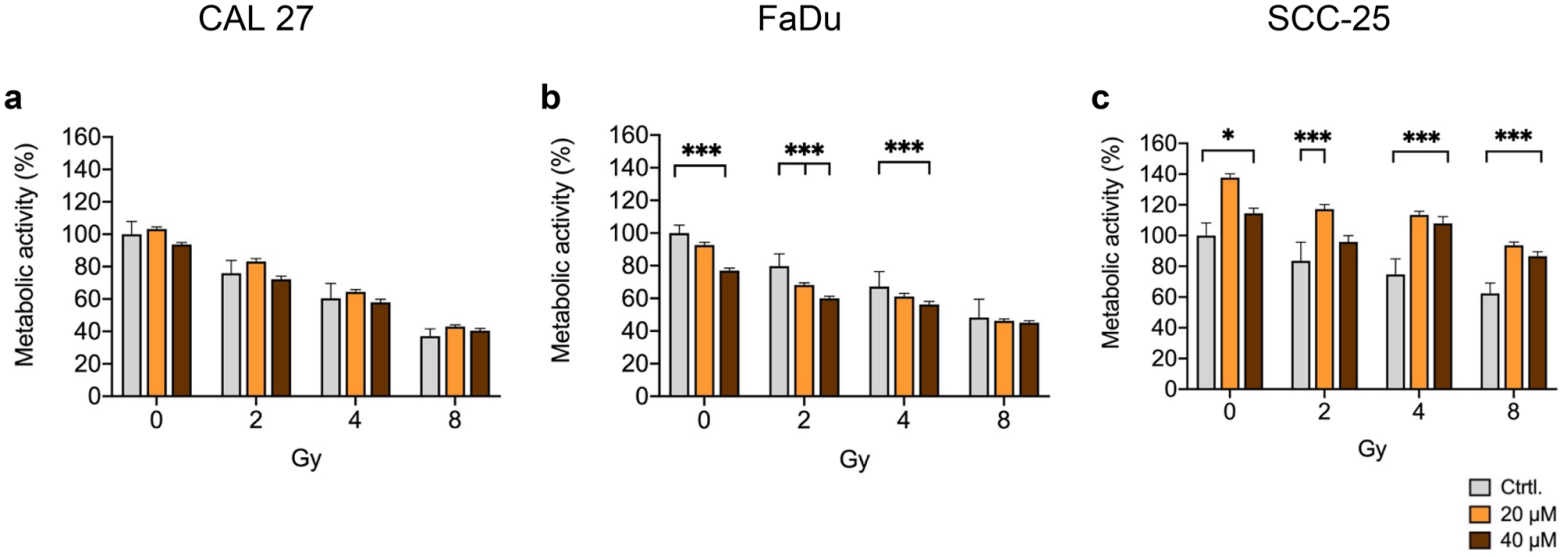
Galunisertib decreased metabolic activity of FaDu cells, while it increased in SCC-25 and was unaffected in Cal27 cells after 72 h of exposure. Combination treatment of radiation with galunisertib did not affect metabolic activity in Cal27, but significantly decreased in FaDu at 2 and 4 Gy. In SCC-25, galunisertib antagonized the effect of radiation on metabolic activity. Bar graphs are mean + SD,* p < 0.05, *** p < 0.001. Data represents metabolic activity as percentage of vector-treated control for three independent experiments including 6 replicates per condition

To determine the effect of galunisertib on metabolic activity, a resazurin-based reduction assay was performed after exposure of HNSCC cell lines to galunisertib for 72 h. Vector (0.16% DMSO) treated cells served as control. Galunisertib showed different effects on metabolic activity in all three tested HNSCC cell lines. While CAL27, a tongue carcinoma cell line, showed no effect (Fig. 1a), FaDu, a hypopharyngeal SCC cell line, showed significantly reduced activity at 40 µM (metabolic activity: 80% vs. 100%, P < 0.001, Fig. 1b). The second tongue carcinoma cell line, namely SCC-25, showed an increased activity in response to galunisertib exposure which was highest at 20 µM, reaching 138%, and declined to 115% at 40 µM compared to control (P < 0.05, Fig. 1c). Radiation single treatment decreased metabolic activity in all three cell lines in a dose-dependent manner, with the strongest effect in CAL27, followed by FaDu and SCC-25 after a radiation dose of 8 Gy (Fig. 1 a-c, all P < 0.001). In combination treatment, galunisertib showed a significant additional reduction of metabolic activity at a concentration of 40 µM in FaDu together with 2 and 4 Gy (60 vs. 80% and 56 vs. 67%, respectively, P < 0.001), but no additional effect could be observed at 8 Gy (P = 0.974). No significant effect of combination treatment could be observed in CAL27. For SCC-25, the increased metabolic activity observed in galunisertib single treatment was also present in combination with radiation, increasing at 8 Gy from 62 to 87% in combination with 40 µM galunisertib (P < 0.001, Fig. 1c).

### Galunisertib reduces HNSCC cell migration

**Fig. 2:**
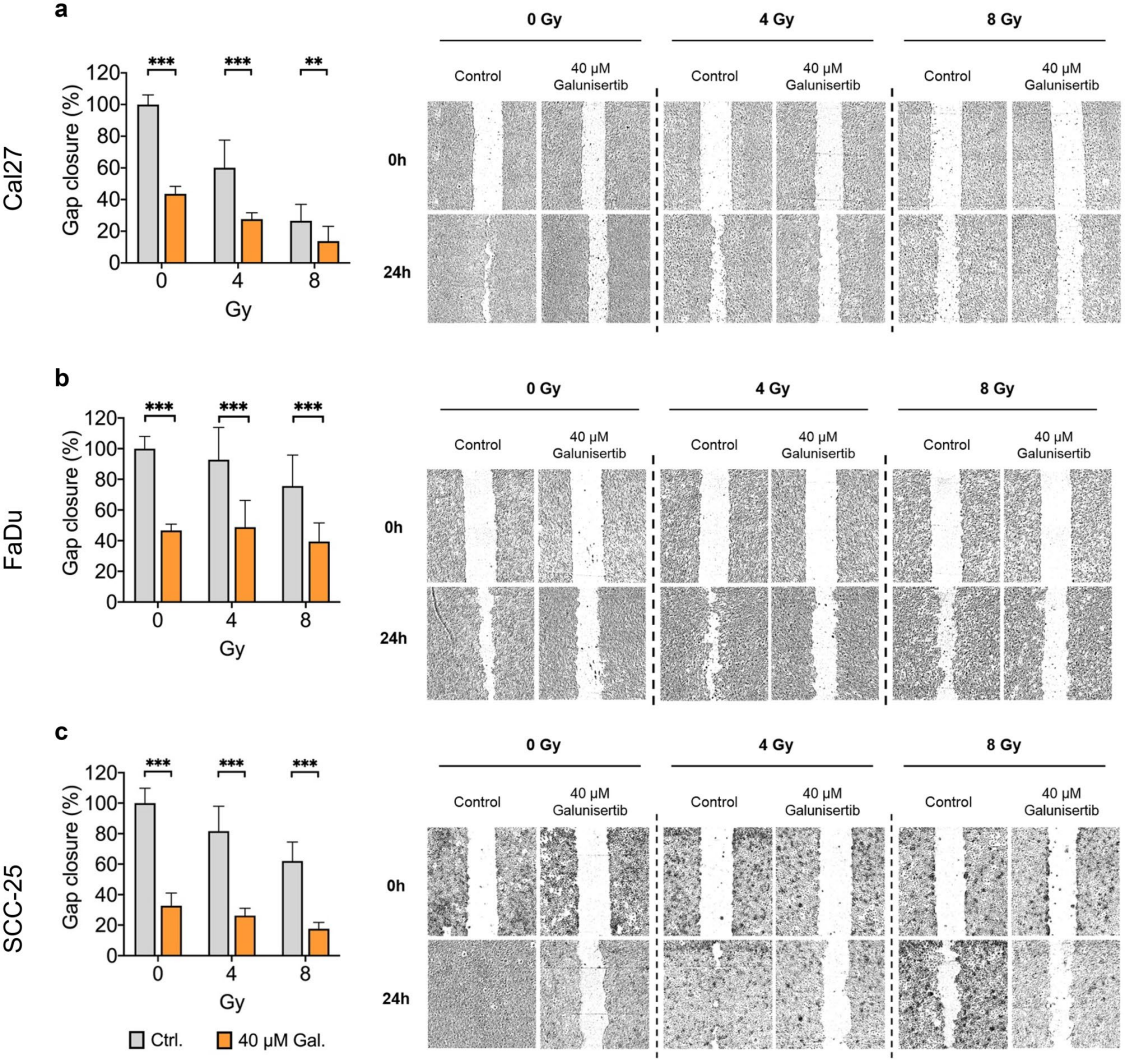
Galunisertib treatment showed an additive effect on cell migration in combination with radiation for CAL27 and SCC-25 (a + c). Bar graphs are mean + SD,** p < 0.01, *** p < 0.001. Data represents gap closure as a percentage of control for three independent experiments including 6 replicates per condition

To study the impact of galunisertib on cell migration, a wound-healing assay was performed as described previously [16]. Galunisertib single treatment inhibited migratory capabilities in all tested cell lines (Fig. 2). Radiation single treatment also reduced cell migration in all cell lines in a dose-dependent manner, with the strongest effect in CAL27, followed by SCC-25 and FaDu. In combination treatment, 40 µM galunisertib showed an additive effect in CAL27 (gap closure at 8 Gy + 40 µM vs. 0 Gy + 40 µM.: 14 vs. 44%, P < 0.001) and SCC-25 (18 vs. 33%, P = 0.015, Fig. 2a,c) while the effect of combination treatment was antagonistic in FaDu (39 vs. 47%, P = 0.882, Fig. 2b).

### Galunisertib reduces clonogenic survival in SCC-25

**Fig. 3:**
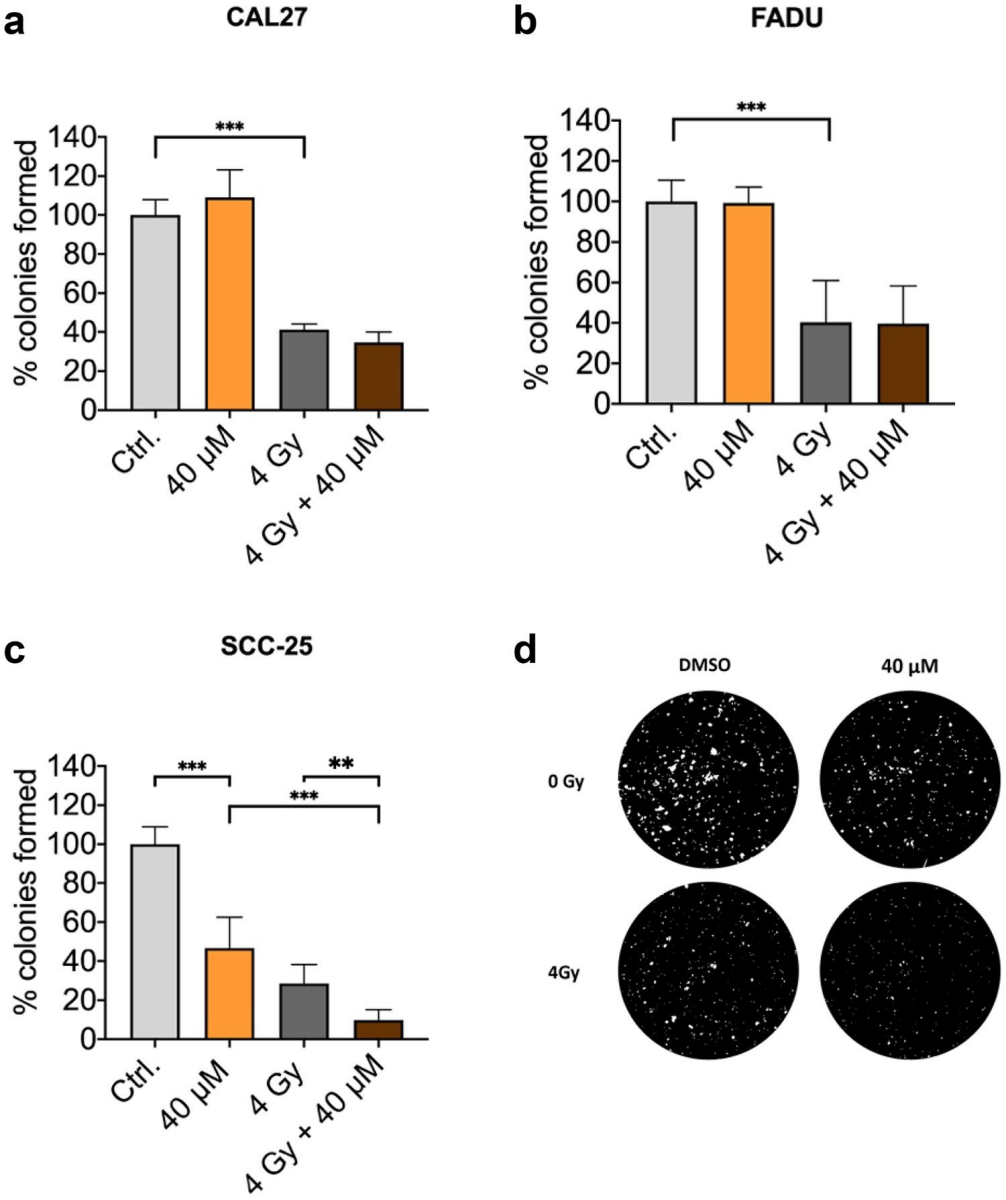
Galunisertib did not affect clonogenic survival in CAL27 or FaDu (a,b), but reduced clonogenic survival in SCC-25 and showed an additive effect in combination with radiation treatment (c). Representative images of the analysis for SCC-25 (d). Bar graphs are mean + SD,** p < 0.01, *** p < 0.001. Data represent the percentage of colonies normalized to control for three independent experiments including three replicates per condition

To test for the effect of galunisertib on clonogenic survival, colony formation assays were performed. Galunisertib single treatment showed a significant reduction in clonogenic survival of SCC-25 cells by 50% (P < 0.001), but did not affect clonogenic survival in CAL27 or FaDu. Radiation single treatment reduced clonogenic survival in all tested cell lines, with the strongest effect in SCC-25 (Fig. 3c). In combination with radiation, an additive effect could be observed for SCC-25 (percent colonies formed at 40 µM vs. 4 Gy + 40 µM, 47 vs. 10%, P < 0.001, Fig. 3. c, d), while no radiosensitizing effect was observed in CAL27 or FaDu (Fig. 3a,b).

### Galunisertib affects cell cycle progression in SCC-25

**Fig. 4:**
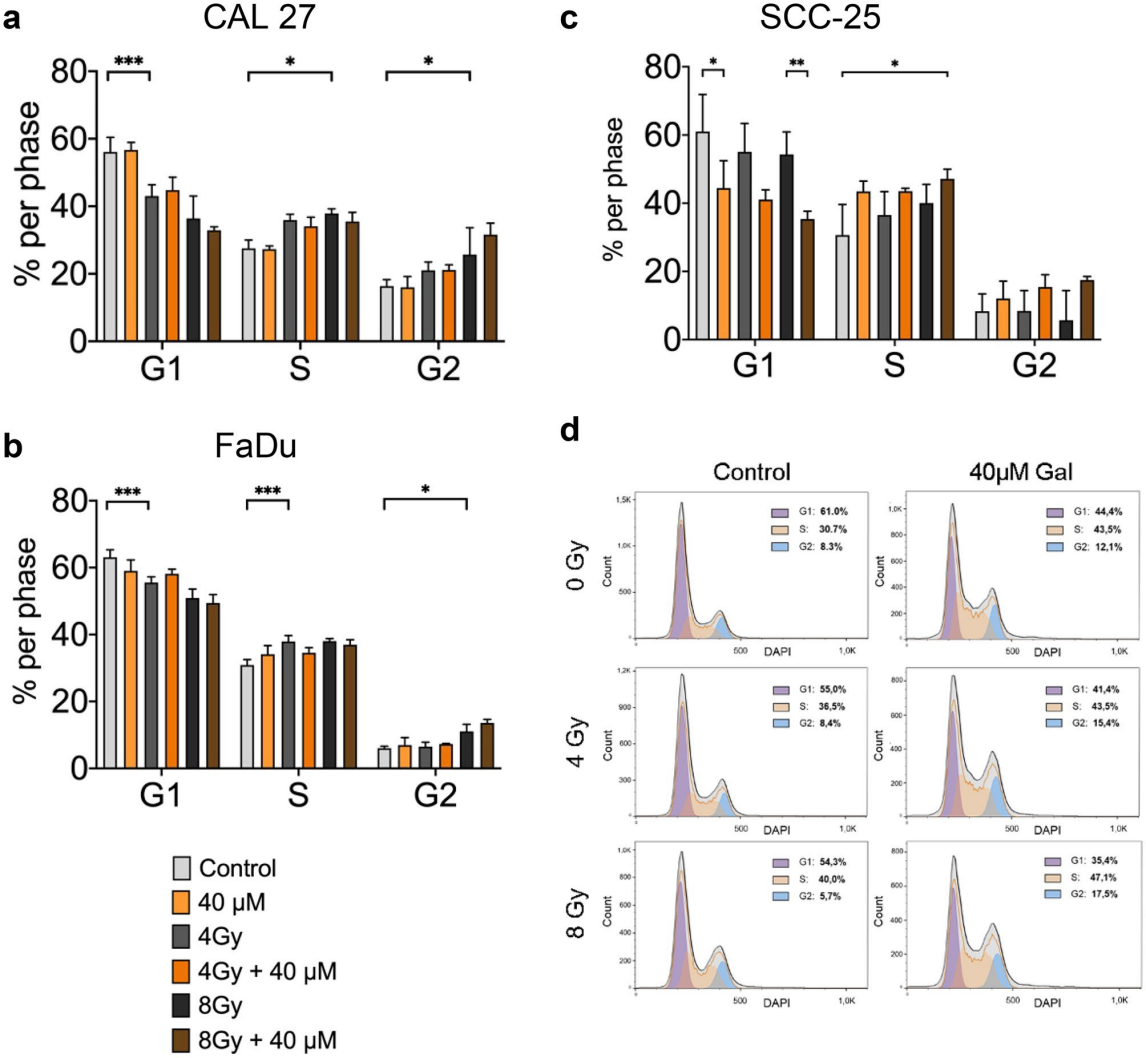
All cell lines showed a shift from G_1_ towards S phase in response to radiation (a,b,c). Galunisertib significantly reduced SCC-25 in G_1_ phase after single and combination treatment and increased cells in S phase in combination with radiation (c,d). Bar graphs are mean + SD,* p < 0.05, ** p < 0.01, *** p < 0.001. Data represent the percentage of cells in each respective cell cycle phase for three independent experiments including three replicates per condition

We used flow cytometry for DAPI-labelled DNA content to investigate the effects of galunisertib adjuvant to radiation on cell cycle progression. Cells were treated for 72 h with either 40 µM galunisertib, radiation or a combination of both. DMSO treated cells served as control. Galunisertib single treatment showed a reduction of cells in G_1_ phase for SCC-25 (Ctrl. vs. 40 µM, 61% vs. 44%, P = 0.019) and a subsequent increase in S and G_2_ phase, although this difference was not statistically significant. Galunisertib single treatment did not affect cell cycle distribution in CAL27 or FaDu. While radiation treatment significantly decreased cells in G_1_ phase in CAL27 (Ctrl. vs. 4 Gy, 56% vs. 36%, P < 0.001) and FaDu (Ctrl. vs. 4 Gy, 63% vs. 51%, P < 0.001) it did not significantly decrease cells in G_1_ phase in SCC-25. However, in combination treatment, an additive effect could be observed in SCC-25, further decreasing cells in G_1_ phase from 54 to 35% (8 Gy vs. 8 Gy + 40 µM respectively, Fig. 4c, P = 0.006).

## Discussion

This is the first study to describe the in vitro effect of galunisertib in head and neck squamous cell carcinoma. Analysing three head and neck squamous cell carcinoma cell lines using standard preclinical assays, we observed varying antineoplastic effects in all tested cell lines at clinically relevant drug concentrations.

Radiation therapy is a cornerstone of treatment in a majority of patients suffering from head and neck squamous cell carcinoma. Radioresistance, therefore, poses a major problem, which could be potentially abrogated using radiosensitizing agents. Research into the molecular mechanisms of radioresistance has led to the identification of many potential targets [17]. Transforming growth factor-beta (TGF-ß) has emerged as such a target [18] and the availability of the small molecule TGF-ß receptor I inhibitor galunisertib, which is currently under clinical investigation as an antineoplastic agent, has led us to investigate its radiosensitizing potential in head and neck squamous cell carcinoma.

Since its first demonstration of antitumor effects in xenograft models of non-small lung cancer and breast cancer [19], galunisertib has progressed through phase I and II clinical trials and is one of the most advanced candidates among small molecule TGF-ß inhibitors [20]. Clinical studies investigating galunisertib in hepatocellular carcinoma [21] and pancreatic cancer [22] have shown improved outcome of the treatment group. Most importantly, various clinical trials reported a safe toxicity profile with no dose-limiting events [20]. Clinical trials in phase I and I/II investigating the combination of galunisertib with radiation treatment in hepatocellular carcinoma (NCT02906397) and malignant glioma (NCT01220271), respectively, are underway. Interestingly, we could not find any in vitro studies that investigated the combination of galunisertib with radiation. However, a study by Hardee et al. has shown increased sensitivity to radiation therapy in glioblastoma treated with the TGF-ß receptor I inhibitor LY364947 [23]. Furthermore, Yang et al. have shown that another TGF-ß receptor inhibitor (LY2109761) increased radiosensitivity in gastric cancer in vitro and in vivo [24], highlighting the expected potential of this treatment combination.

Here, we aimed at investigating the effect of galunisertib at clinically relevant doses. We, therefore, searched the literature for studies investigating the plasma concentration of galunisertib in clinical trials first and found that patients who received a dose of 150 mg twice daily reached a plasma concentration of ~ 10,000 ng/ml (corresponding to ~ 27 µM) [25]. We thus subsequently prespecified to investigate galunisertib at concentrations of 20 and 40 µM, since higher serum concentrations might not be achievable in the clinical setting. Notably, at comparable concentrations of up to 10 µM, galunisertib showed potent and selective inhibition of SMAD 2/3 phosphorylation in hepatocellular carcinoma cells [26] and anaplastic carcinoma cells [27].

We observed the strongest effects of galunisertib in SCC-25. Cell migration and clonogenic survival were significantly inhibited by single treatment and showed additive effects in combination with radiation, suggesting a radiosensitizing antineoplastic effect. Surprisingly, galunisertib treatment had a stimulating effect on metabolic activity, a surrogate marker for cell proliferation, in SCC-25, which also antagonized the inhibitory effect of radiation treatment. We furthermore observed a cell cycle shift with a significant reduction of cells in G_1_ phase. This could potentially be explained by an inhibition of TGF-ß mediated G_1_ phase arrest, which is often lost in malignant cells [28], but appears to be still intact in SCC-25. Indeed, it has been shown that TGF-ß signaling intermediates can remain intact and activate TGF-ß responsive promoters in some HNSCC cell lines [29]. It would appear that the increase in metabolic activity at 72 h could be associated with this effect, emphasizing the dichotomous role of TGF-ß in tumor progression [30]. Interestingly, a recent publication by Oshimori et al. identified TGF-ß-responding SCCs that displayed hallmarks of malignancy during a slow-cycling proliferation. Interestingly, those cells showed better protection against DNA damaging agents [31]. An inhibition of this mechanism might therefore explain more efficient radiation-induced DNA damage. In the other two tested cell lines, drug response was not as distinct. In FaDu, we could observe a reduction in metabolic activity and cell migration after galunisertib treatment. In CAL27, an inhibitory effect could be observed for cell migration after galunisertib treatment with an additive effect with radiation. Consistent with our results, galunisertib showed inhibitory effects on cell migration in primary cholangiocarcinoma cells [32] and hepatocellular carcinoma [26] at comparable concentrations of 50 and 10 µM, respectively. Furthermore, a study by Zhang et al. showed a reduction in cell viability in ovarian cancer cells in vitro, however, at concentrations above 100 µM [33].

Mechanistically, the observed differences between cell lines could be explained by different degrees of TGF-ß escape. Martin et al. have shown that CAL27 harbour an inactivating SMAD4 mutation, which is strictly required for downstream signaling of TGF-ß receptor I [34]. FaDu has been shown to be SMAD4-deficient [35]. Notably, galunisertib has shown high selectivity for the TGF-ß pathway and potent inhibition of TGF-ß mediated SMAD2/3 phosphorylation [36]. This poses the question of the underlying mechanism of observed differences between CAL27 and FaDu. Interestingly, while CAL27 remained insensitive to TGF-ß stimulation after SMAD4 re-expression in a study by Martin et al. [34], suggesting TGF-ß receptor alterations, FaDu showed partial restoration of TGF-ß responsiveness in a study by Hummer et al. [35]. In a subsequent analysis of tissue microarrays for SMAD4 expression, Martin et al. found detectable SMAD4 expression in ~ 82% of HNSCC patients by immunohistochemistry, suggesting that a substantial patient population could potentially benefit from a TGF-ß receptor inhibitor therapy.

Here, we explored the effect of TGF-ß receptor I inhibition in HNSCC monoculture, neglecting the paracrine signaling observed in the tumor microenvironment. However, autocrine TGF-ß potentiation appears to play an important role in tumor progression and could explain the observed effects. [37]. Therefore, our in vitro results cannot be directly applied to predict the response of HNSCC to Galunisertib treatment in vivo. The evident importance of the tumor microenvironment in TGF-ß signaling calls for more advanced cancer models, including cells of the tumor stroma, in future studies [38]. Notably, similar studies investigating the effect of Galunisertib both in vitro and in vivo, found consistent antineoplastic effects [33, 39, 40]. It can therefore reasonably be assumed that the observed effect of Galunisertib on HNSCC cell lines might also be relevant in vivo.

In conclusion, we demonstrated that galunisertib shows significant radiosensitizing and antineoplastic effects in one out of three tested HNSCC cell lines in vitro. This observation correlates with evidence for TGF-ß signaling alterations in the two less responsive cell lines, therefore providing a rationale for further investigation of this new drug.

## Abbreviations

HNSCC: Head and neck squamous cell carcinoma
TGF-ß: Transforming growth factor ß
